# Supplementary material for: A fault-intrusion-tolerant system and deadline-aware algorithm for scheduling scientific workflow in the cloud
Source: PeerJ Comput Sci. 2021 Nov 2;7:e747. doi: 10.7717/peerj-cs.747 (PMC8576554; doi:10.7717/peerj-cs.747)
Supplement: Supplemental Information 6 [file peerj-cs-07-747-s006.zip › Myproject1.1/docs/org/cloudbus/cloudsim/examples/package-summary.html]

org.cloudbus.cloudsim.examples


JavaScript is disabled on your browser.


- Overview
- Package
- Class
- Use
- Tree
- Deprecated
- Index
- Help

- Prev Package
- Next Package

- Frames
- No Frames

- All Classes

# Package org.cloudbus.cloudsim.examples

- Class Summary

  | Class | Description |
  | --- | --- |
  |  |  |
  | --- | --- |
  | CloudSimExample1 | A simple example showing how to create a datacenter with one host and run one cloudlet on it. |
  | CloudSimExample2 | A simple example showing how to create a datacenter with one host and run two cloudlets on it. |
  | CloudSimExample3 | A simple example showing how to create a datacenter with two hosts and run two cloudlets on it. |
  | CloudSimExample4 | A simple example showing how to create two datacenters with one host each and run two cloudlets on them. |
  | CloudSimExample5 | A simple example showing how to create two datacenters with one host each and run cloudlets of two users on them. |
  | CloudSimExample6 | An example showing how to create scalable simulations. |
  | CloudSimExample7 | An example showing how to pause and resume the simulation, and create simulation entities (a DatacenterBroker in this example) dynamically. |
  | CloudSimExample8 | An example showing how to create simulation entities (a DatacenterBroker in this example) in run-time using a globar manager entity (GlobalBroker). |
  | CloudSimExample8.GlobalBroker |  |

- Overview
- Package
- Class
- Use
- Tree
- Deprecated
- Index
- Help

- Prev Package
- Next Package

- Frames
- No Frames

- All Classes
